# Supplementary material for: From sequence to enzyme mechanism using multi-label machine learning
Source: BMC Bioinformatics. 2014 May 19;15:150. doi: 10.1186/1471-2105-15-150 (PMC4229970; doi:10.1186/1471-2105-15-150)
Supplement: Additional file 2 — Java code of ml2db. Additional file ml2db_code.tar.gz contains the Java source code to run the multi-label machine learning experiments and save the results to database. The code’s Javadoc is included. [file 1471-2105-15-150-S2.zip › additional file 2/ml2db/ml2db/doc/main/ml2db/mulan/learn/traintest/TrainTestFullRun.html]

TrainTestFullRun


JavaScript is disabled on your browser.


- Overview
- Package
- Class
- Use
- Tree
- Deprecated
- Index
- Help

- Prev Class
- Next Class

- Frames
- No Frames

- All Classes

- Summary:
- Nested |
- Field |
- Constr |
- Method

- Detail:
- Field |
- Constr |
- Method


main.ml2db.mulan.learn.traintest

## Class TrainTestFullRun

- java.lang.Object
- - main.ml2db.mulan.learn.traintest.TrainTestFullRun

- ---

    

  ```
  public class TrainTestFullRun
  extends java.lang.Object
  ```

  Runs a full train/test evaluation: 1. generate the ARFF file for training and
  the (old) file for testing 2. filter the (old) test arff file by the train
  file 3. generate a .model file containing the serialised classifier trained
  on the train arff 4. train on the train arff and test on the (new, filtered)
  test arff

  Version:
  :   28 Mar 2011

  Author:
  :   Luna De Ferrari ldf(at)st-andrews.ac.uk

- - ### Constructor Summary

    Constructors

    Constructor and Description

    `TrainTestFullRun()`
  - ### Method Summary

    Methods

    | Modifier and Type | Method and Description |
    | --- | --- |
    | `static void` | `fullRun(DbManagers dbs, boolean filtered, boolean serialise, boolean predict, boolean saveTrueNegatives, int train, int test, MulanLearner learner)` Executes: 1. |
    | `static void` | `main(java.lang.String[] args)` |

    - ### Methods inherited from class java.lang.Object

      `equals, getClass, hashCode, notify, notifyAll, toString, wait, wait, wait`

- - ### Constructor Detail


    - #### TrainTestFullRun

      ```
      public TrainTestFullRun()
      ```
  - ### Method Detail


    - #### fullRun

      ```
      public static void fullRun(DbManagers dbs,
                 boolean filtered,
                 boolean serialise,
                 boolean predict,
                 boolean saveTrueNegatives,
                 int train,
                 int test,
                 MulanLearner learner)
      ```

      Executes: 1. filtering the test file by the train file (if filtered =
      false), 2. model training and serialisation, 3. train-test evaluation

      Parameters:
      :   `saveTrueNegatives` -


    - #### main

      ```
      public static void main(java.lang.String[] args)
      ```


- Overview
- Package
- Class
- Use
- Tree
- Deprecated
- Index
- Help

- Prev Class
- Next Class

- Frames
- No Frames

- All Classes

- Summary:
- Nested |
- Field |
- Constr |
- Method

- Detail:
- Field |
- Constr |
- Method
